# Localization of Lipid Droplets in Embryonic Axis Radicle Cells of Soybean Seeds under Various Imbibition Regimes Indicates Their Role in Desiccation Tolerance

**DOI:** 10.3390/plants12040799

**Published:** 2023-02-10

**Authors:** Salma Khanam, Kimie Atsuzawa, Yasuko Kaneko

**Affiliations:** 1 Graduate School of Science and Engineering, Saitama University, Saitama 338-8570, Japan; 2 Comprehensive Analysis Center for Science, Saitama University, Saitama 338-8570, Japan; 3 Department of Natural Science, Faculty of Education, Saitama University, Saitama 338-8570, Japan

**Keywords:** soybean seeds, embryonic axis, radicle cells, desiccation tolerance, lipid droplets, plasma membrane, tonoplast, starch grains

## Abstract

Desiccation tolerance allows plant seeds to remain viable during desiccation and subsequent re-hydration. In this study, we tried to develop an experimental system to understand the difference between desiccation tolerant and desiccation sensitive radicle cells by examining excised embryonic axes after re-desiccation and subsequent imbibition under various regimes. Embryonic axes excised from soybean (*Glycine max* (L.) Merr.) seeds imbibed for 3 h to 15 h which remained attached to the cotyledons during imbibition would grow normally after 24 h of desiccation and re-imbibition on wet filter paper. By contrast, when the embryonic axes excised after 3 h imbibition of seeds were kept on wet filter paper for 12 h to 16 h, their growth was significantly retarded after 24 h of desiccation and subsequent re-imbibition. Numerous lipid droplets were observed lining the plasma membrane and tonoplasts in radicle cells of desiccation tolerant embryonic axes before and after desiccation treatment. By contrast, the lipid droplets lining the plasma membrane and tonoplasts became very sparse in radicle cells that were placed for longer times on wet filter paper before desiccation. We observed a clear correlation between the amount of lipid droplets lining plasma membranes and the ability to grow after desiccation and re-imbibition of the excised embryonic axes. In addition to the reduction of lipid droplets in the cells, a gradual increase in starch grains was observed. Large starch grains accumulated in the radicle cells of those axes that failed to grow further.

## 1. Introduction

Seed desiccation tolerance is the characteristic of orthodox seeds; the term refers to tolerance of repeated dehydration and implies the ability of cells to rehydrate successfully. Seeds of orthodox species gain tolerance of desiccation towards the end of seed maturation; it is generally lost during germination with the onset of radicle elongation. A complex nexus of physiological, genetic, and molecular mechanisms is involved in survival under desiccation in orthodox seeds [1].

At the early stage of germination, orthodox seeds can tolerate repeated desiccation and re-hydration. This characteristic contributes to the survival of germinated seeds under unexpected adverse conditions and is considered an important ecological trait [2], which is exploited in agriculture to produce seeds which can overcome adverse situations [3,4]. In order to improve germination performance under favorable and unfavorable conditions, seed priming is practiced frequently in agriculture [4,5]. This is a method that takes advantage of the desiccation tolerance of germinating seeds: hydration is interrupted during the initial stage of germination before the radicle protrudes, and planting activates a metabolic restart [6]. Among various types of priming technology, the easiest and most economical method is hydro priming involving water uptake for a limited duration after which treated seeds are dehydrated again for storage [6].

During desiccation and hydration of seeds, several types of changes occur inside cells and some of them can be lethal. It is necessary to prevent lethal cellular injury if the seed is to be desiccation tolerant. Hence a tolerant seed must have some distinctive features to accomplish this. At least three main defensive systems have been characterized [1,2]: (i) accumulation of soluble sugars, which stabilize membranes and proteins in dry conditions [7,8] and promote the formation of a glass phase in the cytoplasm [9,10]; (ii) the ability to prevent, tolerate, or repair damage from free radical attack during desiccation [11] and; (iii) the presence of protective LEA proteins that are inducible by ABA [12]. However, there are still many aspects which have to be resolved.

One of the most important cell components which can be severely affected during desiccation and re-hydration is the plasma membrane. Damage to plasma membranes during the re-hydration of seeds has been examined by measuring electrolyte leakage [13,14]. Numerous lipid droplets have been observed lining plasma membranes of mature orthodox seed cells and a relationship with desiccation tolerance has been suggested [13,15,16] The lipid droplets serve not only as storage material to be used as a carbon source but also provide material for the remodeling of plasma membranes [17,18]. Oleosins (lipid body proteins), are found on the surfaces of lipid droplets in all desiccation tolerant plants, but are absent from lipid droplets in the seeds of desiccation sensitive plants [19], suggesting the involvement of lipid droplets in desiccation tolerance.

Recently, several studies have indicated that triacylglycerols (TAG) have a role in the protection of cells under stress conditions. Lipids are the main structural elements of cellular membrane systems, and as such are often the most significant casualties of the adverse impacts of abiotic environmental stresses, including low temperature and desiccation; they suffer first because of their high susceptibility to peroxidation damage and degradation [20,21]. Biological membranes can thus experience severe damage under adverse environmental conditions, and it has been hypothesized that the TAG reservoirs in lipid droplets may be a source of energy and supply building blocks to provide the plant with the required elements for the fast recovery of its membranes and metabolism after stress cessation [22,23].

The major storage reserve substances of the soybean seed are proteins and lipids [9]. The oil content in soybeans has a high proportion of triglycerides and monounsaturated and polyunsaturated fatty acids, such as linoleic and linolenic acids (52.5% and 7.5%, respectively) [24]. Linoleic acid is known to be a constituent of plasma membranes.

As storage materials, starch grains are minor components in soybean seeds, both in cotyledons and embryonic axes; small starch grains are occasionally observed in the plastids of cotyledon cells. Usually only after the emergence of hypocotyl and cotyledons into light, starch grains develop in the chloroplasts of those cells.

We aimed to investigate the relationship between lipid droplets lining plasma membranes and the desiccation tolerance of the cells after repeated desiccation and re-hydration on embryonic axis radicle cells of soybean seeds. Soybean is one of the most important cultivated crops which produce desiccation-tolerant seeds and intact embryonic axes can be excised after 3 h imbibition of seeds. It is known that during germination, imbibed seeds have the ability to survive repeated desiccation and re-hydration only for a limited period and this ability is lost once radicles start elongating [2,25]. When soybean seeds (*Glycine max* (L.) Merr. cv maple arrow) are germinated for a shorter period of time, such as 6 h, before drying, they show dehydration tolerance, which means after re-imbibition the seeds usually grow. If, however, they germinate for a more extended period (36 h), they lose the ability to tolerate desiccation [14]. Pea and maize seeds also lose their desiccation tolerance after between 18 and 24 h, and by 48 h of imbibition before desiccation, respectively [8].

Storage lipids in cotyledons are converted into sugar via the glyoxylate cycle [17] and sucrose is then transported to growing embryonic axes. The embryonic axis cells also contain a certain amount of protein bodies and lipid droplets, enough to ensure growth of the axes to some extent even in the absence of cotyledons. The protein bodies in radicle cells are degraded rapidly and converted into vacuoles upon imbibition. The originally small and numerous vacuoles in a cell eventually merge into one large vacuole and contribute to radicle cell elongation by producing turgor pressure. Among the constituent parts of the seeds, the radicle has been shown to be the most sensitive to re-desiccation after imbibition [26], although the desiccation tolerance of radicle cells can be strengthened by osmotic treatment, such as with PEG [27].

The main purpose of this research is to investigate the role of lipid droplets in keeping soybean seed cells alive during the processes of desiccation and subsequent imbibition under deliberately rather artificial conditions. Embryonic axes were excised from cotyledons in order to eliminate the constant influence and nutrition supply from cotyledons and to focus on cellular events especially in the radicle cells of embryonic axes.

We thus designed an experimental system by which desiccation and re-hydration damage and localization of lipids could be investigated cytologically in radicle cells of embryonic axes. The distribution of lipid droplets lining plasma membranes would seem to indicate a protective function in the maintenance of cell membrane integrity during the processes of desiccation and re-hydration.

## 2. Results

### 2.1. Observation of the Growth of Embryonic Axes after Re-Imbibition

**Figure 1:**
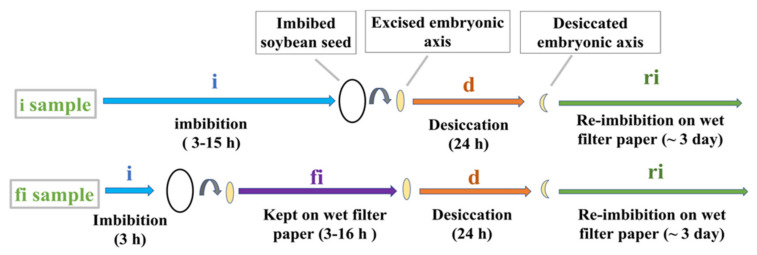
Experimental scheme for sample preparation. Second row: excised embryonic axes from imbibed (**i**) seeds were further imbibed on wet filter paper (**fi**) before desiccation (**d**) and re-imbibition (**ri**).

**Figure 2:**
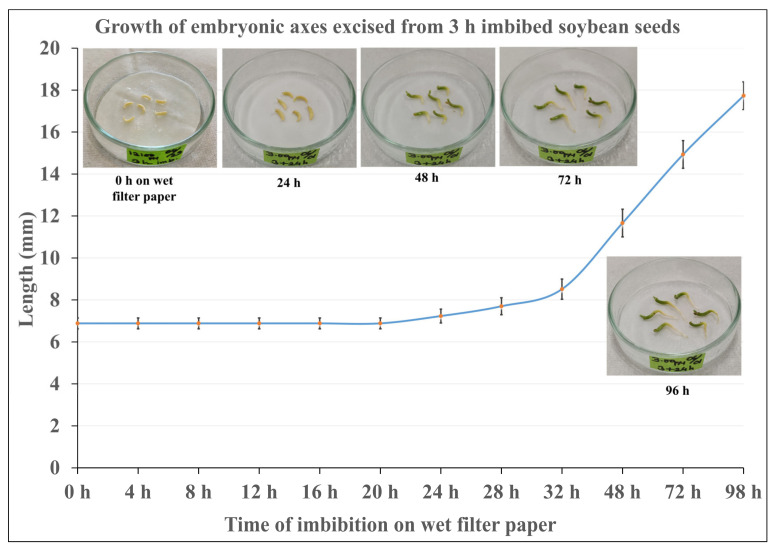
Embryonic axes excised after 3 h imbibition of seeds were kept on wet filter paper for up to 96 h (4 days). The embryonic axes did not grow until 20 h, which corresponds to Phase 2 (lag phase) of germination.

As a framework for the investigation, we established an experimental system (Figure 1) in which embryonic axis growth was gradually retarded after desiccation and re-imbibition in order to identify incremental cytological changes. Since possible damage was evaluated in excised embryonic axes grown on wet filter paper, their growth without desiccation treatment was examined first (Figure 2). Length of the axes did not change until 20 h. Fresh weight of the axes also did not increase up to 20 h. The axes started to elongate after 24 h, and growth continued at least until 96 h. This shows that embryonic axes are able to grow more or less normally without cotyledons for up to 4 days. Since it is known that after the radicle starts elongating, desiccation tolerance of imbibed seeds tends to be lost, incubation on wet filter paper was limited to up to 20 h, a time during which the potential of axes to continue growing is assured if they are not subjected to desiccation.

**Figure 3:**
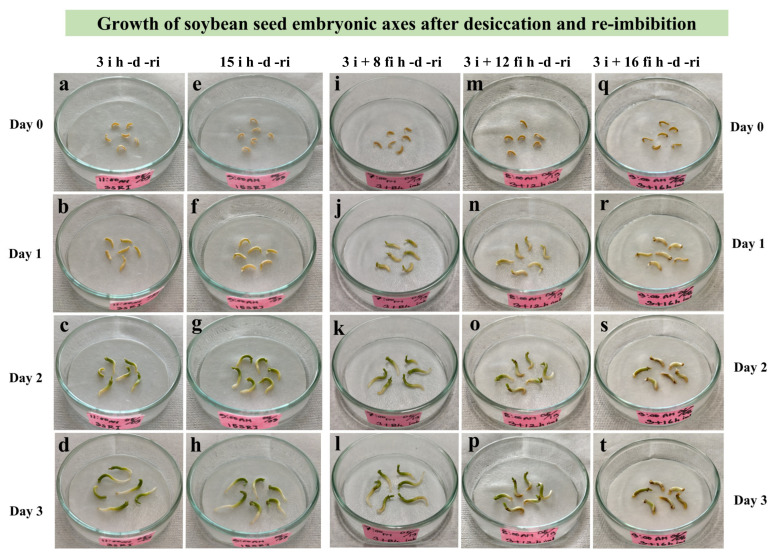
Growth of soybean embryonic axes during 3 days after re-imbibition (**ri**) following 24 h desiccation (**d**). Embryonic axes were excised after 3 h (**a**–**d**), and 15 h (**e**–**h**) imbibition (**i**) of seeds or kept on wet filter paper (**fi**) for an extra 8 h (**i**–**l**), 12 h (**m**–**p**), or 16 h (**q**–**t**) after excision from 3 h imbibed (**i**) seeds. The growth of embryonic axes, especially radicles, was significantly retarded in the samples with long additional incubation on wet filter paper after excision from imbibed seeds (**p**,**s**,**t**).

For the first desiccation experiment, embryonic axes excised from seeds imbibed for different durations (3 to 15 **i** h) were desiccated (**d**) and re-imbibed (**ri**) (Figure 1. **i** sample). All of them grew normally and differences depending on imbibition time were not recognized (Figure 3a–h). Then extra imbibition of excised embryonic axes on wet filter paper was tried (Figure 1. **fi** sample). In the case of the **fi** sample, the growth of embryonic axes after desiccation (**d**) and re-imbibition (**ri**) decreased gradually as they were kept longer on the wet filter paper (8, 12, and 16 **fi** h) (Figure 3i–t).

**Figure 4:**
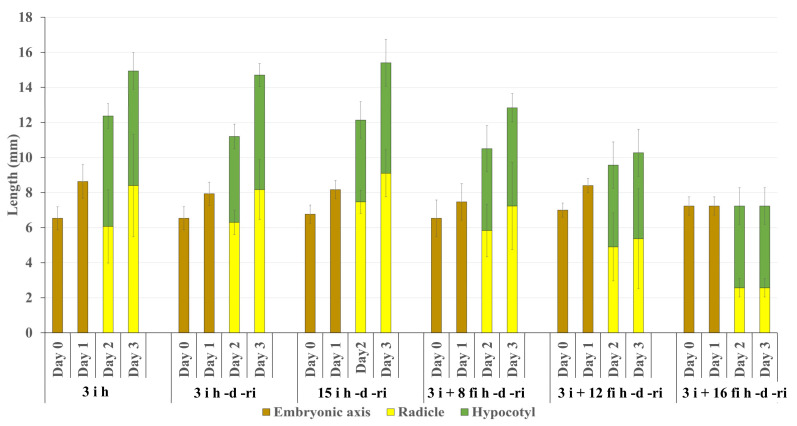
Growth of soybean embryonic axes, hypocotyls, and radicles during 3 days after re-imbibition (**ri**) following 24 h desiccation (**d**). Embryonic axes were excised after 3 h and 15 h imbibition (**i**) of seeds or kept on wet filter paper (**fi**) for an extra 8 h, 12 h, and 16 h after excision from 3 h imbibed (**i**) seeds. As a control, growth of embryonic axes excised after 3 h imbibition and kept on wet filter paper without desiccation is shown at the left. Bars represent the standard deviation (SD).

The length of the axis and its hypocotyl and radicle were compared during growth after desiccation (**d**) and re-imbibition (**ri**). The growth of 3 **i** h (control without desiccation and re-imbibition), and 3 **i** h -**d** -**ri** and 15 **i** h -**d** -**ri**, embryonic axes was similar up to 3 days (Figure 4). By contrast, the growth of axes and especially their radicle portion after desiccation (**d**) and re-imbibition (**ri**) was suppressed gradually in **fi** samples, when they were kept on wet filter paper for longer (8, 12, and 16 **fi** h) before desiccation (**d**) (Figure 4).

### 2.2. Fluorescence Microscopy Observation

**Figure 5:**
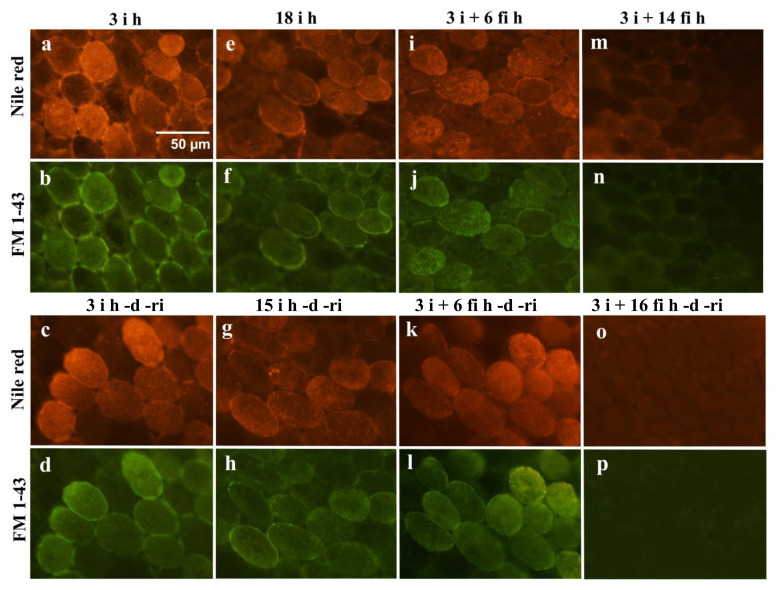
Observation of lipid droplets along the plasma membrane of radicle cells. Lipid droplets and plasma membranes were fluorescently stained with Nile red (**a**,**c**,**e**,**g**,**i**,**k**,**m**,**o**) and FM 1-43 (**b**,**d**,**f**,**h**,**j**,**l**,**n**,**p**). The staining patterns of Nile red (intense fluorescence primarily indicating lipid droplets) was only subtly different from the pattern exhibited by the membrane stain FM 1-43 in radicle cells after 3 h imbibition (3 **i** h: (**a**,**b**)) and after re-imbibition following 3 h imbibition and desiccation (3 **i** h -**d** -**ri**: (**c**,**d**)). Intense Nile red staining along the plasma membrane was also observed in radicle cells after 18 h imbibition (18 **i** h: (**e**,**f**)) and in the embryonic axes kept for 6 h on wet filter paper after excision (3 **i** + 6 **fi** h: (**i**,**j**)). The intense staining remained to some extent after desiccation and re-imbibition of equivalent samples (15 **i** h -**d** -**ri**: (**g**,**h**); 3 **i** + 6 **fi** h -**d** -**ri**: (**k**,**l**)). The Nile red staining along the plasma membrane of the radicle cells was reduced significantly in embryonic axes kept on wet filter paper longer than 12 h after excision (3 **i** + 14 **fi** h: (**m**,**n**)), and Nile red staining in those cells was negligible (3 **i** + 16 **fi** h -**d** -**ri**: (**o**,**p**)) after desiccation and re-imbibition.

Localization of lipid droplets in embryonic radicle cells was compared in differently treated samples. Our intention was to use fluorescence microscopy to show staining for lipid droplets (stained with Nile red) along the plasma membrane (stained with FM 1-43) in imbibed (**i**) and re-imbibed (**ri**) radicle cells of embryonic axes excised from 3 h to 18 h imbibed seeds (Figure 5a–h). We confirmed beforehand that the Nile red fluorescence could not be detected under the conditions for taking images for FM 1-43. However, owing to the similar nature of lipid components constituting lipid droplets and plasma membranes, it may not be possible to separate lipid droplets and plasma membranes clearly with the fluorescent techniques employed. Intense fluorescence of Nile red and FM 1-43 tended to co-localize at portions of plasma membranes. In the case of the embryonic axes kept for longer on wet filter paper (**fi**) after excision from the cotyledons, the intensity of the lipid staining at the cell membrane was gradually reduced before desiccation (**d**) (Figure 5i,j,m,n) and the lipid staining was almost negligible after re-imbibition (**ri**) of the samples kept on filter paper (**fi)** for longer periods (Figure 5o,p).

**Figure 6:**
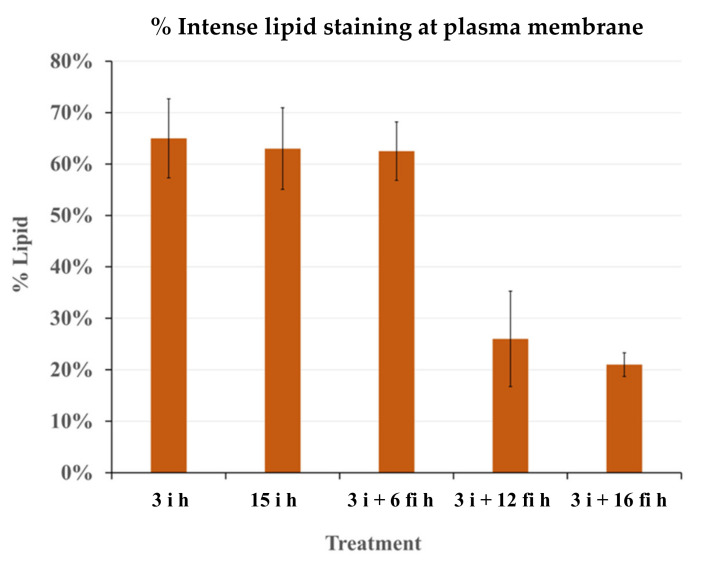
Percentage of the intensely Nile red stained portion of the plasma membrane. Average of five radicle cortical cells in each sample. Bars represent the standard deviation (SD).

The percentage of the length of the intense Nile red stained portion at the plasma membrane was measured and compared (Figure 6). The length of intense Nile red staining indicating lipid droplet accumulation was reduced significantly in the **fi** samples when they were kept on the wet filter paper for 12 h and 16 h.

### 2.3. TEM Observation

**Figure 7:**
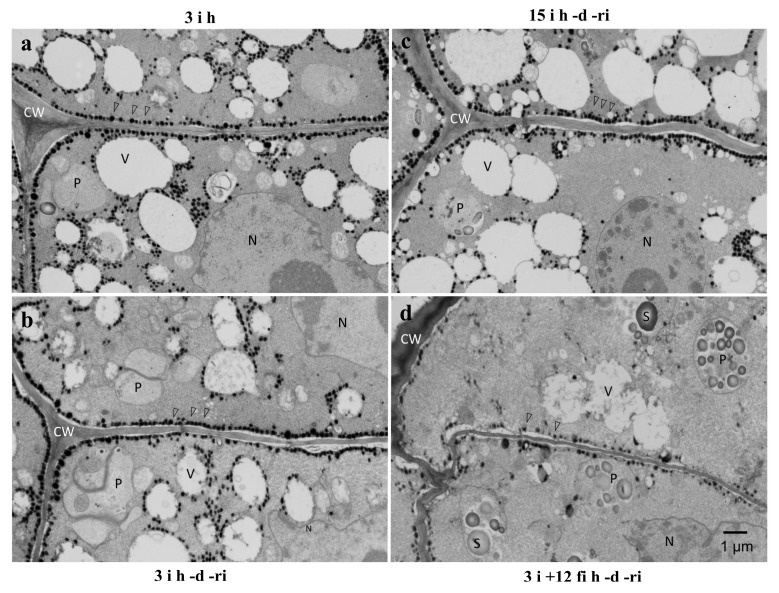
TEM observation of radicle cells in differently treated embryonic axes. Numerous lipid droplets (open arrowheads) were observed lining the plasma membrane of radicle cells of embryonic axes of 3 h imbibed seeds (3 **i** h: (**a**)) and the localization remained after desiccation and re-imbibition of the excised axes (3 **i** h -**d** -**ri**: (**b**)). Lipid droplets were also observed in the vicinity of many small vacuoles (V) in those cells. Similar characteristics were observed in the radicle cells of embryonic axes excised after 15 h imbibition of seeds, desiccated, and re-imbibed (15 **i** h -**d** -**ri**: (**c**)). By contrast, lipid droplets lining the plasma membranes and tonoplasts were reduced significantly in the radicle cells of embryonic axes kept on wet filter paper for 12 h before desiccation and re-imbibition (3 **i** + 12 **fi** h -**d** -**ri**: (**d**)). Instead, numerous starch grains (S) were observed in these cells. CW: cell wall, N: nucleus, P: plastid, S: starch grains, V: vacuole, open arrowheads: lipid droplets.

The ultrastructure of radicle cells of differently treated embryonic axes was observed by transmission electron microscopy (Figure 7). In the radicle cells of embryonic axes of 3 h and 15 h imbibed seeds (3 **i** h, 15 **i** h), numerous lipid droplets were lining the plasma membrane and some lipid droplets in the cytoplasm surrounded small vacuoles or protein bodies (Figure 7a,b). A similar localization of lipid droplets was still observed after re-imbibition (**ri**) following desiccation (**d**) (Figure 7c). However, in the case of embryonic axes kept on wet filter paper for 12 h (**fi**) before desiccation (**d**), the amount of lipid droplets along the plasma membrane and surrounding small vacuoles was reduced significantly. Interestingly, numerous starch grains were observed in plastids inside the radicle cells, instead (Figure 7d).

### 2.4. Light Microscopy Observation

**Figure 8:**
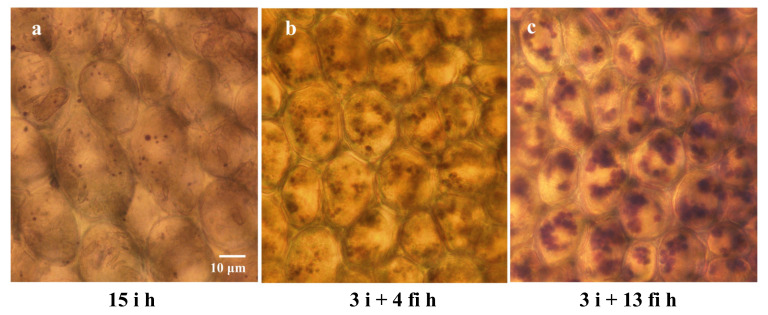
Light microscopy observation of starch grains stained by iodine in embryonic radicle cells after 15 h imbibition of seeds (15 i h: (**a**)), and in the cells of embryonic axes kept 3 h (3 **i** + 4 **fi** h: (**b**)) or 13 h (3 **i** + 13 **fi** h: (**c**)) on wet filter paper after excision.

**Figure 9:**
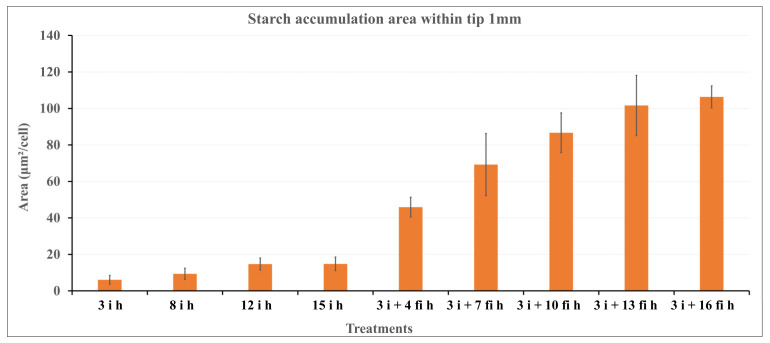
Area occupied by starch (μm²/cell) in the radicle cells of embryonic axes under various treatments. Starch grains per cell increased significantly after excised embryonic axes were placed on wet filter paper. The longer the wet filter incubation time, the more starch grains occupied the cells. Bars represent the standard deviation (SD).

The amount of starch contained in the cells in the differently treated samples was compared. In the embryonic axis radicle cells after 3 h to 15 h imbibition (3 **i** h, 8 **i** h, 12 **i** h, 15 **i** h), starch grains were very sparse and only small starch grains were observed (Figure 8a). A substantial amount of starch in numerous grains was observed in the radicle cells of embryonic axes kept on wet filter paper (**fi**) after excision from the imbibed seeds (Figure 8b,c). A gradual increase of starch content per cell was obvious, as shown in Figure 9.

## 3. Discussion

Orthodox seeds acquire desiccation tolerance towards the end of seed maturation, concomitant with the accumulation of storage materials, such as protein bodies and lipid droplets [28]. Since many orthodox seeds have the ability to tolerate repeated desiccation and re-hydration for a limited duration after imbibition of the seeds [25], the vigorous growth after desiccation and re-imbibition of embryonic axes excised from 3 h to 15 h imbibed seeds in this experiment was not unexpected. Since our aim was to establish an experimental system to observe the relationship between cytological change, especially lipid droplet localization, and loss of desiccation tolerance, further trials were conducted to determine conditions which ensure reduction of vigor after desiccation and re-imbibition. The prolonged imbibition of excised embryonic axes on wet filter paper fulfilled this purpose. The longer the imbibition on wet filter paper, the less growth of the axes after desiccation and re-imbibition was observed.

The obvious reason for the clear difference between imbibition with cotyledons and excised axes without cotyledons is that stored nutrition is continuously transported to the embryonic axes during prolonged imbibition of whole seeds, whereas excised axes are obliged to use their own storage material for survival. Major storage materials in soybean seeds are proteins in protein bodies and lipids in lipid droplets. Lipids in cotyledons are metabolized to sugar via the glyoxylate cycle for transportation [17]. It should be noted that under our experimental regimes, that is, during extra imbibition of excised axes on wet filter paper for up to 18 h, the axes hardly grew at all: the length of the axes were about the same as before desiccation. We confirmed that embryonic axes excised from imbibed seeds could grow on wet filter paper for 4 days and would start elongating at 24 h (Figure 2).

We thus obtained an experimental system in which desiccation tolerant and desiccation sensitive states could be clearly distinguished. Dehydration injury of germinating soybean seeds may manifest as loss of germination capacity, slower growth rates of isolated axes or hypocotyl, or root curling [14]. Our system looked at embryonic axes and radicle cells. Extra imbibition on wet filter paper (Figure 1) for up to 16 h of excised embryonic axes from imbibed seeds resulted in retarded growth after dehydration and re-imbibition (Figure 3 and Figure 4). We found that the growth of the radicle, in particular, was reduced in proportion to the duration of the extra imbibition. After 16 h of extra imbibition, no growth of the embryonic axes was observed and after 12 h imbibition, growth of the radicle was suppressed to about half of that of samples without extra imbibition on wet filter paper. Our observations imply that embryonic axes imbibed on wet filter paper for 12–16 h after excision should be considered desiccation sensitive, whereas embryonic axes after 3–15 h (total imbibition time) are desiccation tolerant.

The most desiccation sensitive part of the seed is known to be the radicle. At the first step of germination, the radicle cells are expected to go through drastic change in preparation to elongate promptly. In our experimental system, apparent damage was observed at the radicles of embryonic axes imbibed for 16 h on wet filter paper prior to desiccation and re-imbibition.

Since growth of the radicle was most affected after long extra imbibition on wet filter paper, the cells in this portion were observed by various microscopic techniques. Abundant lipid droplets were observed lining plasma membranes in desiccation tolerant embryonic axes, whereas lipid droplets at the plasma membrane were reduced dramatically in desiccation susceptible embryonic axes (Figure 5 and Figure 7). Lipid droplets lining plasma membranes have been reported in embryonic axis cells in various seeds, such as maize [13,16], pea [29,30], and soybean [31]. Their presence has been presumed to be associated with vigorous growth during germination, because some of the most severe damage is assumed to occur at the plasma membrane during desiccation and rehydration [13,16], and the lining of lipid droplets should have an important role in protecting the membranes. The importance of lipid droplets lining the plasma membrane to maintain desiccation tolerance was shown clearly in our experimental system. Lipid droplets were also observed lining or in the vicinity of small vacuoles which must have been converted from protein bodies during the early phase of imbibition in desiccation tolerant embryonic axes in this study (Figure 7). Since the priority of radicle cells during germination is to elongate promptly, enlargement of vacuoles must be essential. These lipid droplets may also be protecting membranes, in this case the vacuolar membranes (tonoplasts), to ensure the growth of the cells.

In fluorescent microscopy, the staining patterns of Nile red and FM 1-43 were in many cases very similar, though not identical. Because the lipid components of plasma membranes and lipid droplets are very similar, it may not be possible to clearly distinguish lipid droplets and plasma membranes by the methods used here. However, the staining patterns of the dyes do indicate localization of lipid components, including lipid droplets along membranes and suggest a close relationship between them. Especially in the radicle cells of desiccation tolerant embryonic axes, lipid droplets were almost always attached to either plasma membranes or vacuolar tonoplast membranes by electron microscopy. Tonoplasts are critical to ensure the subsequent enlargement for cell elongation. It must thus be worthwhile to protect and support especially these membranes during desiccation and re-hydration.

When lipid droplets and plasma membranes were fluorescently stained and observed in radicle cells of embryonic axes treated differently, we observed a clear relationship between the intensity of lipid droplets lining plasma membranes and desiccation tolerance. Lipid droplets lining plasma membranes in seed cells have been observed frequently in various species, such as maize [13,16], pea [29,30], and soybean [31]. Since some of the most sensitive parts of the cell during desiccation and re-hydration are the plasma membranes, special protection mechanisms to ensure recovery of the intact membranes should exist. In transmission electron microscopy of rapidly frozen embryonic axis plumules of pea seeds, in which a more native state of the cell structure could be captured, lipid droplets lining plasma membranes at 6 h after initiation of imbibition were mobilized to surround developing plastids, possibly with the aid of ER membranes and actin filaments after 16 h of imbibition [30]. Lipid droplets lining plasma membranes often appear to fuse with the plasma membrane and interact with each other. It is also known that the lipid droplets contain materials for remodeling plasma membranes [17,18]. Certain proteins surrounding lipid droplets exist only in desiccation tolerant seed cells [19], which further indicates the involvement of lipid droplets in the desiccation tolerance of the membranes.

Unexpectedly, accumulation of starch grains was observed in the desiccation susceptible embryonic axes radicle cells (Figure 8 and Figure 9). The number of starch grains and amount of starch per cell increased gradually during imbibition of excised embryonic axes. Because the nutrition supply from cotyledons was interrupted by excision of the embryonic axes, some metabolic alteration must have occurred to provide an energy source for the further growth of the excised embryonic axes since they could not rely on carbon supplied by cotyledons. Since lipid components gradually decreased during this period, it is conceivable that products of the lipid degradation may have been converted into starch. In general, seed storage lipid is converted into sugar through the glyoxylate cycle involving glyoxysomes [32]. Some glyoxysomes with attached lipid droplets were observed in desiccation susceptible radicle cells by electron microscopy in our system. A similar pathway may have been induced in these cells resulting in the dramatic reduction of lipid droplets which otherwise would protect plasma membranes during desiccation and re-imbibition. Further investigation is necessary to identify the cause and mechanism of the starch synthesis in these embryonic axis cells after excision from cotyledons.

## 4. Materials and Methods

### 4.1. Preparation of Embryonic Axes

Embryonic axes of soybean (*Glycine max* (L.) Merr.cv. Shiratori) seeds were excised after 3 h to 15 h imbibition in tap water with aeration at 23 °C (imbibition step: **i**). Excised embryonic axes after 3 h imbibition of seeds were kept on wet filter paper in Petri dishes for further imbibition for up to 18 h at 23 °C (extra imbibition on filter paper: **fi**). For desiccation of the sample, the imbibed embryonic axes were kept on dry filter paper and left for 24 h at 23 °C (desiccation step: **d**). For re-imbibition of the desiccated samples, they were put on wet filter paper at 23 °C, 16 h light/8 h dark condition (25 μmol/m^2^s at light condition) for up to 3 days to observe the growth of the axes (re-imbibition step: **ri**).

### 4.2. Processing for Fluorescence Microscopy

Free hand sections were cut from within the 1 mm tip portion of the radicles with a razor blade. Both lipid droplets and plasma membranes were stained with Nile red and FM 1-43 simultaneously for 30 min. The concentrations of FM 1-43 and Nile red were 10 µg/mL and 0.5 µg/mL, respectively. The sections were rinsed three times in distilled water and observed with a Nikon ECLIPSE 50i microscope using the TRITC filter set to visualize Nile red and the FITC filter set to visualize FM 1-43 staining.

The fluorescence indicating lipid droplets and membranes often co-localized in this study. Since weak Nile red fluorescence for neutral lipids can be detected with the FITC filter which was used to detect FM 1-43 fluorescence, we carefully eliminated this possibility. Prior to double staining, samples were stained separately and examined by each filter. Since FM 1-43 fluorescence was extremely strong in our staining protocol, we used ND filters to reduce intensity for capturing images. Under these conditions, no signal of Nile red was detected.

### 4.3. Processing for Transmission Electron Microscopy

The samples were cut with a razor blade and fixed with 2% glutaraldehyde in 0.05 M potassium phosphate buffer (pH 6.8) for 2 h at room temperature and at 4 °C overnight. They were rinsed with the same buffer and post-fixed in 2% OsO4 in 0.05 M potassium phosphate buffer (pH 6.8) for 2 h at room temperature, dehydrated in an acetone series and embedded in Spurr’s resin. Ultrathin sections (silver–gold) were stained with aqueous 2% uranyl acetate for 10 min followed by lead citrate for 5 min and observed with a Hitachi H-7500 TEM at an accelerating voltage of 80 kV.

### 4.4. Processing for Light Microscopy

Free hand sections were cut from within the 1 mm tip portion of the radicles with a razor blade. The sections were stained with iodine solution for 10 min, rinsed in distilled water and observed by light microscope (Nikon ECLIPSE 50i). Estimation of the starch occupied area per cell was performed using Image J.

## 5. Conclusions

Continued imbibition of excised embryonic axes from imbibed soybean seeds affected radicle cells and caused gradual loss of desiccation tolerance. Abundant lipid droplets lining the plasma membrane appeared to be gradually reduced and this reduction was accompanied by an increase of starch grains in the radicle cells. The consequent dramatic decrease in lipid droplets lining plasma membranes corresponded with the loss of desiccation tolerance.

The strong correlation between the presence of lipid droplets and desiccation tolerance, persistent under the artificial conditions in our experiments, strengthens the case for a significant role for lipid droplets in the protection of plasma membranes during desiccation and re-hydration.

## Data Availability

All datasets generated for this study are included in the article and further inquiries can be directed to the corresponding author.
